# Enhancing the depth perception of DSA images with 2D–3D registration

**DOI:** 10.3389/fneur.2023.1122021

**Published:** 2023-02-08

**Authors:** Xiaofeng Zhang, Yongzhi Deng, Congyu Tian, Shu Chen, Yuanqing Wang, Meng Zhang, Qiong Wang, Xiangyun Liao, Weixin Si

**Affiliations:** ^1^Shenzhen Institute of Advanced Technology, Chinese Academy of Sciences, Shenzhen, China; ^2^Department of Cardiovascular Surgery, Shanxi Clinical Medical Research Center for Cardiovascular Disease, Shanxi Institute of Cardiovascular Diseases, Shanxi Cardiovascular Hospital, Shanxi Medical University, Taiyuan, China; ^3^Department of Cardiovascular Surgery, Union Hospital, Tongji Medical College, Huazhong University of Science and Technology, Wuhan, China; ^4^Shenzhen Second People's Hospital, Shenzhen, China; ^5^Guangdong Provincial Key Laboratory of Computer Vision and Virtual Reality Technology, Shenzhen Institutes of Advanced Technology, Chinese Academy of Sciences, Shenzhen, China

**Keywords:** 2D–3D registration, weighted similarity measure function, multi-resolution fusion optimization strategy, pyramid convolution, treatment of cerebrovascular diseases

## Abstract

**Objective:**

Today, cerebrovascular disease has become an important health hazard. Therefore, it is necessary to perform a more accurate and less time-consuming registration of preoperative three-dimensional (3D) images and intraoperative two-dimensional (2D) projection images which is very important for conducting cerebrovascular disease interventions. The 2D–3D registration method proposed in this study is designed to solve the problems of long registration time and large registration errors in 3D computed tomography angiography (CTA) images and 2D digital subtraction angiography (DSA) images.

**Methods:**

To make a more comprehensive and active diagnosis, treatment and surgery plan for patients with cerebrovascular diseases, we propose a weighted similarity measure function, the normalized mutual information-gradient difference (NMG), which can evaluate the 2D–3D registration results. Then, using a multi-resolution fusion optimization strategy, the multi-resolution fused regular step gradient descent optimization (MR-RSGD) method is presented to attain the optimal value of the registration results in the process of the optimization algorithm.

**Result:**

In this study, we adopt two datasets of the brain vessels to validate and obtain similarity metric values which are 0.0037 and 0.0003, respectively. Using the registration method proposed in this study, the time taken for the experiment was calculated to be 56.55s and 50.8070s, respectively, for the two sets of data. The results show that the registration methods proposed in this study are both better than the Normalized Mutual (NM) and Normalized Mutual Information (NMI).

**Conclusion:**

The experimental results in this study show that in the 2D–3D registration process, to evaluate the registration results more accurately, we can use the similarity metric function containing the image gray information and spatial information. To improve the efficiency of the registration process, we can choose the algorithm with gradient optimization strategy. Our method has great potential to be applied in practical interventional treatment for intuitive 3D navigation.

## 1. Introduction

In the surgical navigation treatment of cerebrovascular diseases, an accurate identification of the lesion location is one of the important factors that determine the operation plan. Improving the level of multimodal data analysis and image processing technology is an important step in surgical navigation treatment.

Preoperative 3D scanning images can visualize the lesion and provide 3D data information, but they cannot be obtained in real time during surgery, while 2D images cannot provide an accurate 3D navigation information. If both 2D and 3D images are aligned, it can provide an accurate navigation information and improve the success rate of surgery. Multimodal 2D–3D image registration is the key technology. Image registration is the integration of image information gathered from different times or conditions. The goal of image registration is to find the conversion relationship between different images at the same location to obtain an integrated information of two images (1–3).

**Figure 1:**
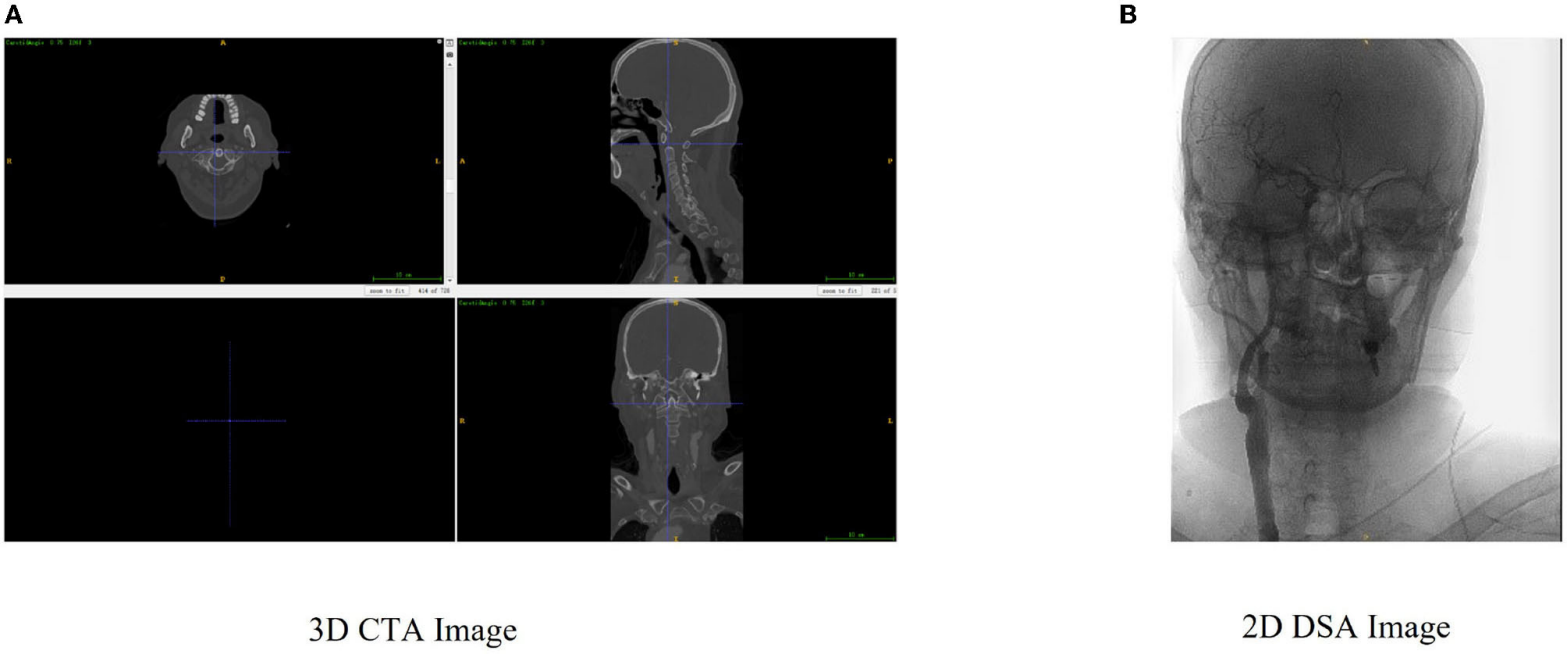
A comparison of three-dimensional computed tomography angiography (3D CTA) image and two-dimensional digital subtraction angiography (2D DSA) image presentation, where **(A)** 3D CTA image is a floating image, and **(B)** 2D DSA image is the reference image.

Multimodal image registration plays an important role in clinical nursing, mainly by including computed tomography angiography (CTA) and digital subtraction angiography (DSA), shown as the CTA image in Figure 1A and DSA image in Figure 1B. In the whole 2D–3D registration process, two types of images, namely, the floating image and the reference image, are required as data sources (4), and the spatial position transformation operation of floating image is required to align it with the reference image to complete the registration (5). However, the registration accuracy of both CTA images and DSA images is low at present, while the time spent on registration is long, which may reduce the surgical efficiency.

At present, for evaluation of the results of the 2D–3D image registration, the selected similarity measurement function includes only the gray-level information of a single image and lacks the spatial information of the image as an aid, resulting in large errors in the results of the final similarity evaluation, such as Normalized Mutual (NM) and Normalized Mutual Information (NMI) (6, 7). In the 2D–3D registration process, the selected optimization algorithm considers only the optimal value and it does not consider improving the registration efficiency, such as Powell and Gradient Descent (GD) (8, 9).

We propose a weighting function with normalized mutual information and gradient difference, considering the grayscale and spatial information of the image, and applying Regular Step Gradient Descent (RSGD) as a means to find the optimal value of the optimization function, reducing the step size by changing the direction of each iteration, avoiding the local optimal value, and improving the speed of registration. The multi-resolution strategy used in this study is the Gaussian pyramid convolution algorithm (10). After the input image is convolved by a layer of convolution, it is sent to the Gaussian pyramid for multi-resolution sampling processing. The pyramid algorithm samples according to the resolution of the input image, and divides the whole sampling process into coarse resolution and fine resolution. The image resolution is the degree of quantization of the image in the horizontal and vertical directions, refers to the degree of image detail that can be shown (11–14). Low resolution image is used to analyze larger structures or the overall content, while high resolution image is used to analyze detailed characteristics. A multi-scale strategy is used to observe continuous images at different scales which helps to understand the image content (15, 16). By applying a Gaussian kernel for multi-scale changes (17), the multi-resolution strategy proposed in this study can enhance the robustness of the algorithm and improve the registration efficiency.

This study makes the following contributions.

1. The 2D–3D registration method proposed in this study optimizes the efficiency of the registration process and the accuracy of the registration results compared to those of the traditional registration methods.

2. We propose normalized mutual information-gradient difference (NMG) as a weighting similarity metric function for the registration results, which includes both grayscale and spatial information of the images.

3. The proposed method chooses multi-resolution fused regular step gradient descent (MR-RSGD) as an optimization algorithm strategy to avoid inefficiency and the local optimal value of the registration result. The proposed method divides the coarse-resolution and fine-resolution images into two sampling processes. Regular step gradient descent (RSGD) can find the optimal value to avoid falling into local extremes, and the result of the objective function is kept constant as a condition for stopping the iterative optimization.

## 2. Related work

In multimodal image registration, the manual manipulation of 2D–3D registration is often limited by the amount of time and expertise required to register segmented data to an image. To be able to reduce the errors introduced by subjectivity, a new intensity-based 2D–3D registration method is proposed, which can exploit all forms of visual information, including not only variations in image intensity but also the apparent contours of structures. However, it uses only information about the grayscale values of pixels without taking into account their surface characteristics (18). A method is proposed that allows to pre-compute all time-intensive steps, entwining spatial information from 3D volumes and 2D projections.

The main work in the actual registration task is reduced to a simple resampling of pre-computed values, which can be performed on the graphics processing unit (GPU), but requires a 3D Radon transformation of the input image, which leads to an increase in computational effort, is also susceptible to image distortion, and can only be applied if the object undergoes a planar to flat transformation (19). A globally optimal method is proposed that iteratively searches the transformation space, constraining the objective function at each stage and discarding parts of the transformation space for which no solution is possible, but only for points, lines, or a combination of both (20).

A new probabilistic 2D–3D vessel registration method that extends the Gaussian Mixture Model (GMM) to 2D–3D registration and integrates orientation information throughout the registration framework is proposed. The final evaluation obtained is based on the 2D projection error, but the out-of-plane orientation error estimate is much larger due to the planar setting (21). A comprehensive landmark-based 2D–3D registration method that uses X-ray images of the object and a 3D reference model to reconstruct the object's 3D pose is proposed. The aforementioned method incorporates an automated 3D landmark extraction technique and a deep neural network for 2D landmark detection, but considers only the limb of one animal sample, ignoring the presence of muscle and other types of soft tissue in other real animal models, complicating the 2D landmark detection, performing noise simulations that do not take into account the model of the X-ray source the response of the detector (22).

The 2D–3D registration based on a convex optimization procedure is applied to a 3D central model of a coronary artery with a pair of perspective images, and the proposed optimization procedure jointly optimizes the correspondence between points and projections, as well as the relative transformations. However, the ability to include translations through centrality is limited, the design of a fast solver for the convex procedure is not considered, and the exact recovery is guaranteed only when there is no noise in the image (23). Biplane 2D–3D registration was used to measure the 3D joint motion, relying primarily on an automated 2D–3D registration procedure based on computed tomography (CT) and magnetic resonance imaging (MRI) images used together to quantify joint motion, but the study was limited to passive shoulder motion and the accuracy of the registration method was limited by the bone quality, speed of motion, and shoulder orientation in the biplane fluoroscopic field of view, and therefore could not be generalized to motion with significant deviations in this area (24).

The use of 2D–3D catheter-based continuous wayfinding fusion of preoperative or intraoperative images in liver surgery can add relevant information to navigated procedures, and the proposed real-time registration of 3D rotational angiographic images in the operative period with intraoperative single-plane 2D fluoroscopic images can improve the guidance of surgical interventions; however, this method deals only with 2D image catheters and the accuracy of the results is low (25).

The choice of the similarity measure function plays an important role in the accuracy of the final result. The statistical method mutual information (MI) (26, 27), which uses the joint probability distribution images to measure the strength of statistical relationships between images, is well adapted to images of different modalities and is therefore used widely in multimodal medical registration. When using mutual information as a similarity metric in the registration framework, mutual information can show a high performance if the recovered displacement is small and it is difficult to obtain a larger displacement, which highlights the advantages of fast global optimization of mutual information. To enhance the fast computation of mutual information and its robustness, many improved algorithms based on the MI have been proposed, introducing normalized mutual information processing (NMI) for sensitivity overlap (28, 29), optimizing the computation of joint distribution, and combining with other intensity-based similarity metrics to form a new metric (30). However, the mutual information calculates only the gray value of each pixel of the image and does not take the spatial features into account, so the spatial information is missing, resulting in a slightly lower registration efficiency.

After choosing the similarity metric function, the choice of optimization algorithm also affects the results of final registration. Powell is mostly used for a local search of optimal values, which has the features of simple calculation, fast convergence, and high accuracy, but it is easy to fall into the local optimal point when searching for the registration parameters, resulting in an insufficient accuracy of the registration. GD can reduce the iterative optimization time to iterate in the direction of the fastest change of the objective function value (31), which can improve the speed of the algorithm, but it does not guarantee to find the global optimal solution that meets the requirements because of the limitation of each iteration step (32–34).

## 3. Methods

**Figure 2:**
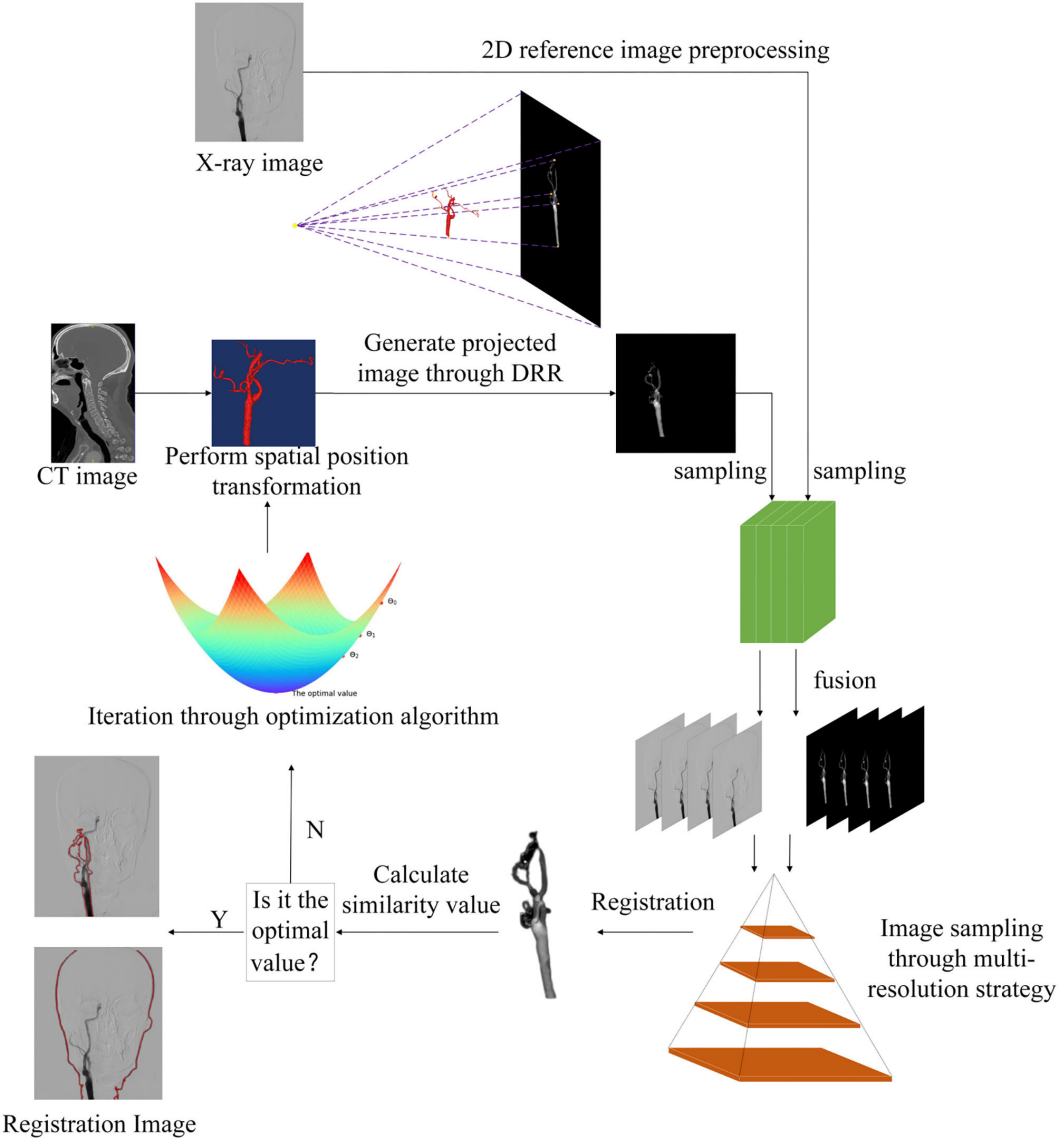
The framework of the proposed two-dimensional three-dimensional (2D–3D) registration method, from the very first input 3D image and 2D image, 3D image is projected to generate 2D image digitally reconstructed radiograph (DRR), 2D image and DRR are processed by pyramidal multi-resolution, then the optimization algorithm regular step gradient descent (RSGD) finds the optimal value of similarity measure function to attain the best registration effect.

The essence of 2D–3D registration is the mathematical problem of iteratively solving the optimal value of an objective function, first adjusting the spatial position of the 3D floating image, then projecting the 3D floating image to generate the 2D projection image, then sampling the projection image and the reference image by multi-resolution strategy, and relying on the optimization algorithm to iteratively find the optimal value, so that the similarity between the two images is maximized, as shown in the 2D–3D registration flowchart in Figure 2.

In the process of 2D–3D registration, we need to first solve the problem of different dimensions of the registration image, using a projection algorithm to project the 3D preoperative image to produce a 2D projection image, the process is the digitally reconstructed radiograph (DRR) algorithm, so that the whole 2D–3D registration is converted to 2D–2D registration. Unlike the shadow formed by ordinary light, the DRR technology is a rendering operation for 3D images, which are converted according to the formation principle of X-ray images. By performing the DRR operation on a 3D object, the corresponding projection image of the object can be obtained, also called the DRR image. The process of DRR generation is shown in Figure 2.

### 3.1. The normalized mutual information-gradient difference

In the 2D–3D image registration process, different similarity metric functions have different abilities to capture image information, and the registration results are dependent on factors such as image morphology, tissue class, and feature information. The proposed function in this experiment is a weighted normalized mutual information-gradient difference metric function.

To improve the accuracy of the registration, this study improves on the two aforementioned similarity metric functions by assigning a coefficient to each of them and combining them to form a new similarity metric function called the NMG, which combines the information of NMI and GD, so that it can take advantage of the normalized mutual information in multimodal image registration and complement the lack of gradient information. Its definition is shown in Equation (1) in Appendix.

The α_1_ and α_2_ of Equation (1) in Appendix are the weights of NMI and GD, respectively, and their sum is 1. In this experiment, α_1_ and α_2_ are both taken as 0.5. Both mutual information (MI) and normalized mutual information (NMI) are based on image information, thus building statistical models based on discrete random variables, and MI represents the information common between two variables. It evolved from Shannon's entropy (Shannon's entropy) theorem (35, 36), while normalized mutual information is a further improvement over the former. Mutual information is widely used in image registration, especially in multimodal image registration. The Shannon entropy formula for image A is shown in Equation (2) in Appendix.

The joint entropy formula for image *A* and image *B* is shown in Equation (3) in Appendix.

The *A* and *B* in all Appendix Equation are pixels in the two images, the *P*(*A*_*i*_) and *P*(*B*_*j*_) of Equation (2) in Appendix are edge probability distributions, and the *p*(*A*_*i*_, *B*_*j*_) of Equation (3) in Appendix is a joint probability distribution. The mutual information is expressed, as shown in Equation (4) in Appendix.

The normalized mutual information makes a change based on mutual information by removing the joint entropy of the images by the sum of the Shannon entropy of the two images. It is defined as shown in Equation (5) in Appendix.

Gradient difference (GD) is to first obtain the gradient images of two images separately, then perform the gradient image subtraction operation to obtain the difference image, and use the difference image to measure the degree of similarity. GD judges the final registration result by measuring the change of low-frequency gray value due to gradient reduction. The formula is shown in Equations (5)–(7) in Appendix.

In Equations (5)–(7) in Appendix, the $\frac{dA}{dm}$ and $\frac{dB}{dm}$ represent the gradient images in the horizontal direction of the two images, $\frac{dA}{dn}$ and $\frac{dB}{dn}$ represent the gradient images in the vertical direction of the two images, *I*_*dV*_ is the difference between the horizontal gradient images, *I*_*dH*_ is the difference between the vertical gradient images, *s* is the scale constraint factor, σ_*v*_ and σ_*h*_ are constants.

### 3.2. Multi-resolution fusion optimization strategy

The 2D–3D registration requires adjusting the position of the 3D image to generate the corresponding projection image in each iteration, which leads to an excessive computation of the registration. To reduce the time consumed in the whole registration process, a multi-resolution strategy can be incorporated into the registration process. The multi-resolution strategy divides the registration process into two, to realize the process from coarse to fine registration, which can improve the probability of finding the optimal solution in the optimization iteration, accelerate the convergence speed of the algorithm, and reduce the registration time loss.

The basic idea of multi-resolution is that the multi-resolution strategy arranges the images in order of their resolutions and aligns them from the lowest to the highest resolution, and the result of the last registration is used as the initial value of the next one. This bottom-up registration method can render the low-resolution images smooth during the registration process, thus improving the robustness of the images, avoiding the algorithm from converging badly in the iterative process and falling into the local optimum, which greatly improves the efficiency and stability of the registration algorithm, speeds up the registration optimization speed, and improves the capture range. In this study, the Gaussian pyramidal convolution algorithm is used for the multi-resolution processing of images, so that the fused multi-resolution pyramidal convolution algorithm improves the stability of registration and registration efficiency. The principle behind the algorithm is that the layer *g*_*k*_ image is convolved from the layer *g*_*k*−1_ image through two convolutional layers characterized by different Gaussian kernels. The computational formula is given in Equation (9) in Appendix.

In Equation (9) in Appendix, *g*_*k*_(*i, j*) denotes the layer *k* image, *i* and *j* denote the number of rows and columns of the image, and *W*(*m, n*) is a window function.

**Figure 3:**
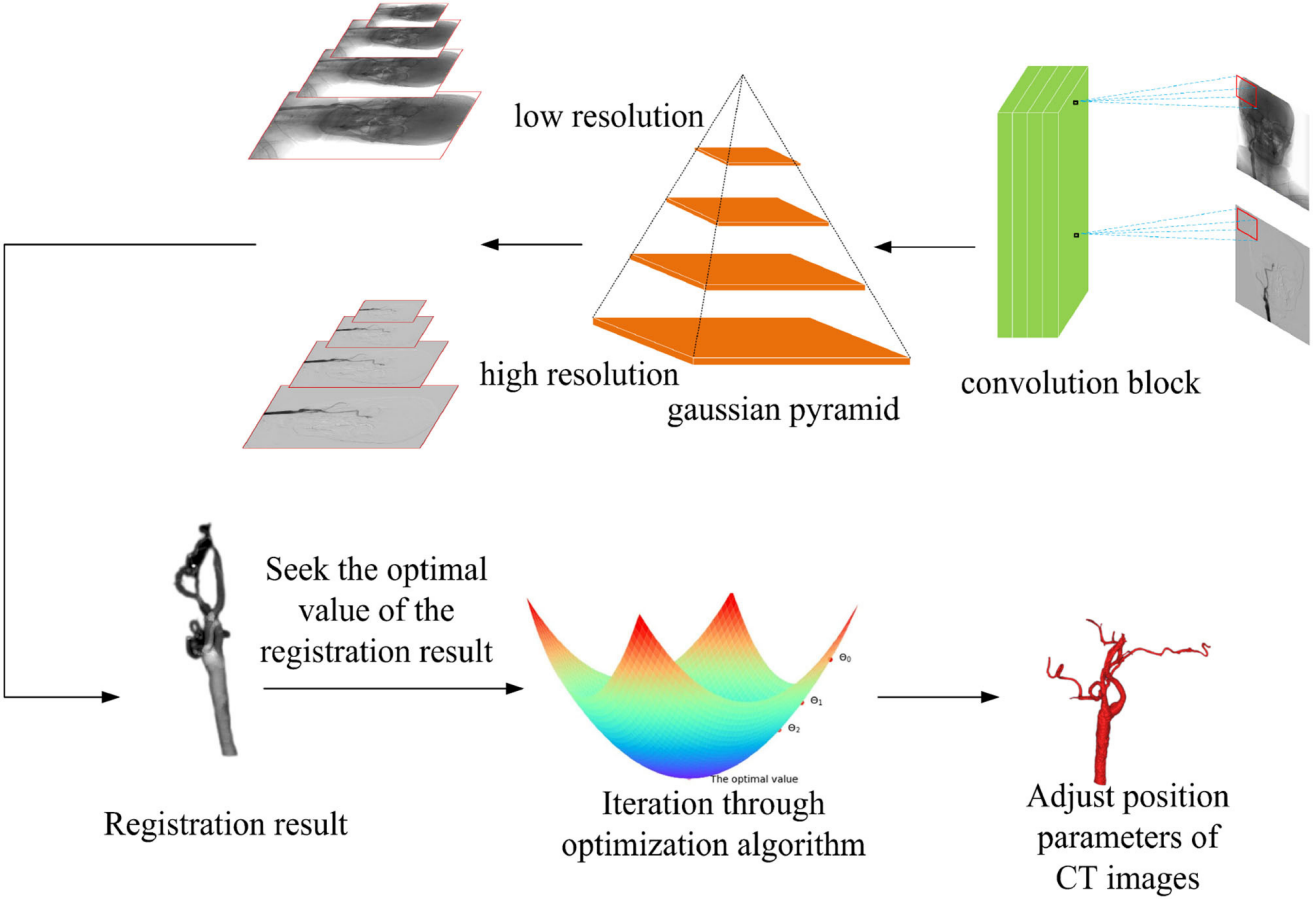
Multi-resolution fusion optimization strategy samples the input image, according to the different image resolutions, and the registration process is divided into two types from coarse resolution to fine resolution. The regular step gradient descent (RSGD) algorithm performs iterative optimization of the registration through the registration results and continuously adjusts the computed tomography (CT) image position parameters to match the optimal projection position, so as to obtain the optimal value of the registration result.

After the similarity metric function of the registration is determined, a suitable optimization algorithm is needed to solve the optimal value of the objective function, so the selection of the optimization algorithm affects the final result of the registration directly. Compared with the traditional gradient descent algorithm, in this experiment, the RSGD algorithm is used to reduce the step size when the gradient direction is changed to update the parameter values each time, it can prevent the generation of excessive step sizes during the gradient descent process, and the solution along the gradient descent direction can improve the registration speed. In each iteration, RSGD takes a fixed step along the gradient direction of the metric and solves for the parameter values within this gradient range for that step value condition. The optimization algorithm formula is given in Equation (10) in Appendix. The optimization process is shown in Figure 3.

In Equation (10) in Appendix, θ_*k*_ denotes the parameter value updated in the iteration of *k* times, θ_*k*+1_ denotes the parameter value updated in the iteration of *k* + 1 times, *k* denotes the number of iteration times, α_*k*_ denotes the step size chosen in the gradient direction in the iteration of *k* times, and *d*_*k*_ times represent the gradient searched in the iteration of *k* times.

In the whole optimization algorithm, the process of finding the optimal value of the similarity measure function, the optimization method adopted is the multi-resolution strategy fused with the conventional stepwise gradient descent algorithm to solve, the pyramid convolution algorithm first samples the coarse-resolution image and the fine-resolution image in two processes from coarse to fine, so as to speed up the alignment efficiency. The similarity metric function finds the metric value and the spatial location parameter, so as to analyze whether the obtained value is the optimal value. Then, the similarity metric function is used to obtain the metric values and spatial location parameters to determine whether the acquired value is the optimal value, if not, the target value is iteratively obtained using the conventional stepwise gradient descent algorithm to speed up the registration, and the optimal value is approximated finally. The algorithm code about this process is shown in Algorithm 1.

**Algorithm 1:** Pyramid convolutional fusion regular step gradient descent (RSGD) optimization process.

**Input:** Moving image, Fixed image;
**Output:** Spatial location parameters and similarity measure values for registration results;
1: convolution(Moving image, Fixed image,kernel);
2: next bold resolution image ⇐ Bold resolution moving image A * kernel;
3: next fine resolution image ⇐ Fine resolution moving image B * kernel
4: pyup(moving image A, dst_A, size),pydown(moving image B, dst_B, size)
5: NMG(dst_A, fixed_A), *NMG*(dst_B, fixed_B); $r_{x},r_{y},r_{z},t_{x},t_{y},t_{z}⇐\{\begin{array}{c} \left(fixed_{A}-dst_{A}\right)\\ \left(fixed_{B}-dst_{B}\right) \end{array}$;
6: After multi-resolution sampling, the calculation of the target value of the similarity measure function is started.
7: $NMI\left(A_{m},A_{f}\right)=1+\frac{MI\left(A_{m},A_{f}\right)}{H\left(A_{m},A_{f}\right)}$;
8: $NMI\left(B_{m},B_{f}\right)=1+\frac{MI\left(B_{m},B_{f}\right)}{H\left(B_{m},B_{f}\right)}$;
9: $GD\left(A_{m},A_{f}\right)=\underset{m,n}{\sum }\frac{\sigma_{v}}{\sigma_{v}+{\left(I_{dV}\left(m,n\right)\right)}^{2}}+\underset{m,n}{\sum }\frac{\sigma_{h}}{\sigma_{h}+{\left(I_{dH}\left(m,n\right)\right)}^{2}}$;
10: $GD\left(B_{m},B_{f}\right)=\underset{m,n}{\sum }\frac{\sigma_{v}}{\sigma_{v}+{\left(I_{dV}\left(m,n\right)\right)}^{2}}+\underset{m,n}{\sum }\frac{\sigma_{h}}{\sigma_{h}+{\left(I_{dH}\left(m,n\right)\right)}^{2}}$;
11: *NMG* = α_1_ · *I*_*NMI*_ + α_2_ · *I*_*GD*_;
12: **while** r(fixed image - moving image)(*r*_*x*_, *r*_*y*_, *r*_*z*_), t(fixed image - moving image)(*t*_*x*_, *t*_*y*_, *t*_*z*_)is not 0 **do**
13: **if** *metric* is not *optimal* **then**
14: α ⇐ 0;
15: *d* ⇐ decrease *d*_*m*_;
16: **while** α < α_*min*_, *d* < *d*_*m*_ **do**
17: *d* ⇐ *f*(*NMG*)′;
18: *NMG* ⇐ *NMG* − α*d*;
19: **end while**
20: **else**
21: Output registration results
22: **end if**
23: **end while**
24: return spatial Location parameter *r*_*x*_, *r*_*y*_, *r*_*z*_, *t*_*x*_, *t*_*y*_, *t*_*z*_& *Metric* *value*

## 4. Results

In the whole registration process, first, the 3D floating image entering the registration is spatially transformed and its position parameters are continuously adjusted to get the best projection position, the projection image is projected to get the qualified projection image, and the 2D reference image is pre-processed at the same time. Second, the Gaussian pyramid convolution algorithm is used for multi-resolution processing, and both the projection image and reference image are sampled from coarse to fine resolution. This process not only improves the signal-to-noise ratio of the sampled signals and the robustness of the algorithm but also increases the speed of registration by compressing the sampling information at each point. The similarity of the sampled projection and reference images is evaluated by the similarity metric function, the optimization algorithm iteratively solves the optimal value of the objective function, until the global optimum is obtained, and the registration process is completed. The first set of data used in this experiment pertains to blood vessel data in a human body, and the data sampled from the CT images and DRR images are shown in Table 1.

**Table 1:** The parameters associated with the first set of cerebrovascular data, the size and resolution of the input three-dimensional computed tomography angiography (3D-CTA) images and two-dimensional digitally reconstructed radiograph (2D DRR) images.

**Data**	**Size**	**Resolution**
CT image	512 × 512 × 726	0.3828 × 0.3828
Analog X-ray image (DRR)	730 × 941 × 1	0.38 × 0.38

As regards the translation of the initial position in CT, rotation values are specifically taken as 0 in the X-axis rotation angle, 0 in the Y-axis rotation angle, 0 in the Z-axis rotation angle, 10 mm for the initial value of translation in the X-axis direction, –50 mm for the initial value of translation in the Y-axis direction, and –70 mm for the initial value of translation in the Z-axis direction, *r*_*x*_ = 0, *r*_*y*_ = 0, *r*_*z*_ = 0, *t*_*x*_ = 10 mm, *t*_*y*_ = –50 mm, and *t*_*z*_ = –70 mm. Table 2 shows the solutions using the first set of data under the same NMG with the application of three different optimization algorithms, Powell, GD, and RSGD. From Table 2, we can see that the registration result error and registration time of the Powell algorithm are larger than those of the other two algorithms, and the registration result error of both GD and RSGD is 0.

**Table 2:** Angle deviation, position deviation, and metric value of the image registration result of blood vessel data.

	**Powell**	**GD**	**RSGD**
Rotation amount along X/(°)	1.1183°	0	0
Rotation amount along Y/(°)	0.0018°	0	0
Rotation amount along Z/(°)	0	0	0
Translation amount along X/(mm)	0	0	0
Translation amount along Y/(mm)	0.2361	0	0
Translation amount along Z/(mm)	0	0	0
Registration time/(s)	61.9249	58.7534	56.5500
Metric value	1.9538	0.0520	0.0037

**Figure 4:**
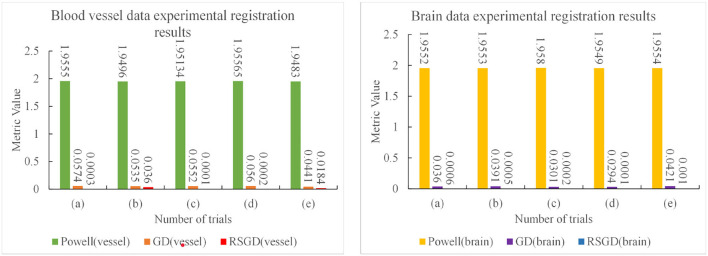
Five sets of controlled experiments were done for the first set of vascular data under three optimization algorithms, Powell, gradient descent (GD), and regular step gradient descent (RSGD). Change the five times position space parameters of the input image, respectively. The first column represents the input digital subtraction angiography (DSA) image, the second column represents the digitally reconstructed radiograph (DRR) image generated by the Powell algorithm, the third column represents the DRR and DSA registration generated by the Powell algorithm, the fourth column represents the DRR image generated by the GD algorithm projection, the fifth column represents the DRR and DSA registration maps generated by the GD algorithm projection, and the results show that some error still seems to be present, the sixth column shows the DRR image generated by the RSGD algorithm, and the seventh column shows the DRR and DSA registration maps generated by the RSGD algorithm, which shows that the registration error is reduced and the registration accuracy is improved compared with the previous two algorithms. The registration results of vessel obtained by Powell in the third column and by GD in the fifth column have errors between the floating image and the reference image, as indicated by the blue arrows.

The experimental results indicate that the accuracy of the registration result can be improved by applying the gradient optimization algorithm to the improved similarity measure function, until the registration error of the spatial parameters tends to 0. Moreover, the time used by RSGD is significantly smaller than that of than the other two optimization algorithms. In summary, RSGD is significantly better than the other two optimization algorithms in iterative optimization of the similarity measure function to find the optimal value. The final experimental results show that the iterative optimization of NMG by applying the RSGD algorithm improved the efficiency and time of registration, and improved the stability, robustness, and accuracy of registration. For two sets of test data, the registration values obtained under different optimization algorithms are shown in Figure 4.

**Figure 5:**
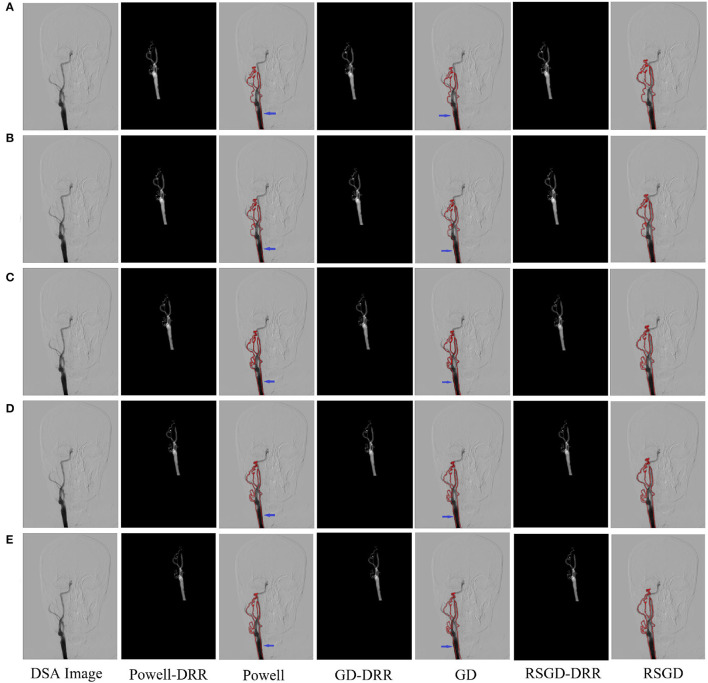
Five controlled trials of three optimization algorithms on blood vessel data and brain data. The labels in row **(A–E)** represent the tests conducted under different initial positions of 3D CTA images. The three initial rotation position parameters of the first group are 0, 0, 0, and the three translation position parameters are 10, −50, −70. Then the remaining five groups can change the measurement value of 20 each time based on the data of the first group. The first column represents the original DSA image, the second, fourth and sixth columns represent the DRR image generated under different optimization algorithms, and the third, fifth and seventh columns represent the registration results of the generated DRR image and DSA image.

The three sets of similarity metric values under the three different optimization algorithms are shown in Table 3. It can be visualized that under the RSGD algorithm optimization, not only is the registration time the fastest, but also the final similarity metric value is also the smallest, and the registration accuracy is the highest. To verify that the RSGD based on the multi-resolution strategy proposed in this study to find the optimal value of NMG has better registration efficiency and stronger robustness, controlled trials were carried out by changing the parameters of the initial position, and the experimental results are shown in Figure 5.

**Table 3:** The parameters associated with the second set of brain data, the size and resolution of the input three-dimensional computed tomography angiography (3D-CTA) images and two-dimensional digitally reconstructed radiograph (2D-DRR) images.

**Data**	**Size**	**Resolution**
CT image	512 × 512 × 675	0.3828 × 0.3828
Analog X-ray image (DRR)	730 × 941 × 1	0.38 × 0.38

To verify the advantages of the weighting function and the optimization algorithm proposed in this study, two controlled experiments of three optimization algorithms (Powell, GD, and RSGD) under three similarity metric functions (MI, NMI, and NMG) were conducted on two sets of data, the blood vessels and the brain, and the comparative experimental results are shown in Figure 4, analyzed by the qualitative and quantitative results obtained from the aforementioned sets of experiments, and the registration method proposed in this study shows certain advantages in registration efficiency and registration accuracy.

To verify that the registration method proposed in this experiment has stronger robustness and can adapt to images of different parts of the human body and different image styles, the second set of brain data was selected for the experiment, and the relevant data are shown in Table 3.

After conducting the second set of data experiments, it can be seen from Table 4 that the conclusions drawn are consistent with the conclusions of the first set of data experiments, the RSGD algorithm for iterative optimization of NMG, the accuracy of the experimental results obtained, and the registration time spent is the best performing set. The results of the Powell algorithm for registration have larger errors in rotation angle and translation distance compared to the other two optimization algorithms, and the registration time used is larger than those of the other two optimization algorithms. The values of the rotation angle error and translation distance error for both the GD and RSGD algorithms are zero, and the registration time used for the RSGD algorithm is smaller than that of the GD algorithm. The RSGD proposed in this experiment is applied to the NMG registration method, and the results of the registration experiments are good, with the distance error of each group being 0, the angle error being 0, the least time consumed, and the error does not increase due to the selection of different parts and different image styles, indicating that the algorithm proposed in this experiment has better generalization applicability and stronger robustness.

**Table 4:** Angle deviation, position deviation, and metric value of image registration result of blood vessel data.

	**Powell**	**GD**	**RSGD**
Rotation amount along X/(°)	1.6180°	0	0
Rotation amount along Y/(°)	0.2304°	0	0
Rotation amount along Z/(°)	0.3820°	0	0
Translation amount along X/(mm)	0.1988	0	0
Translation amount along Y/(mm)	0	0	0
Translation amount along Z/(mm)	0.0157	0	0
Registration time/(s)	53.5833	53.0519	50.8070
Metric value	1.9522	0.0562	0.0003

**Figure 6:**
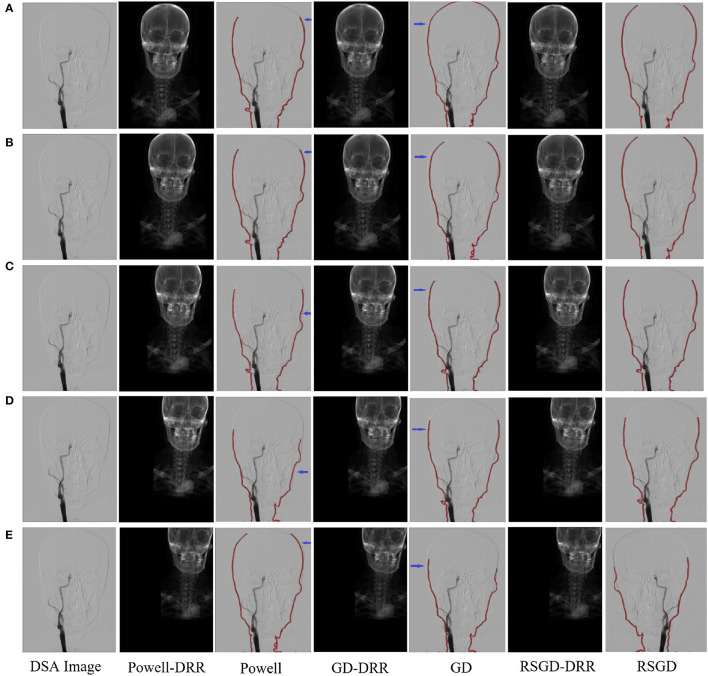
Five sets of controlled trials were done for the second group of brain data under three optimization algorithms, Powell, gradient descent, and regular step gradient descent (RSGD). Change the five times position space parameters of the input image, respectively. The first column represents the input digital subtraction angiograph (DGA) image, the second column represents the digitally reconstructed radiograph (DRR) image generated by the Powell algorithm, the third column represents the DRR and DSA registration generated by the Powell algorithm, the fourth column represents the DRR image generated by GD algorithm projection, the fifth column represents the DRR and DSA registration maps generated by the GD algorithm projection, and the results show that some error still seems to be present, the sixth column shows the DRR image generated by the RSGD algorithm, and the seventh column shows the DRR and DSA registration maps generated by the RSGD algorithm, which shows that the registration error is reduced and the registration accuracy is improved compared with the previous two algorithms, the registration efficiency is also improved. The registration results of the brain obtained by Powell in the third column and by GD in the fifth column have errors between the floating image and the reference image, as indicated by the blue arrows. The labels in row **(A–E)** represent the tests conducted under different initial positions of 3D CTA images. The three initial rotation position parameters of the first group are 0, 0, 0, and the three translation position parameters are 10, −50, −70. Then the remaining five groups can change the measurement value of 20 each time based on the data of the first group.

The results of the second set of data experiments are shown in Figure 6. The registration similarity metric values for the second set of data are shown in the Figure 6, and according to the experimental results, it can be seen that the registration results under the RSGD optimization algorithm are more accurate than the other two. The same transformation of the initial position parameters is performed for the second set of data to verify the universality and stability of the registration framework in this article.

**Figure 7:**
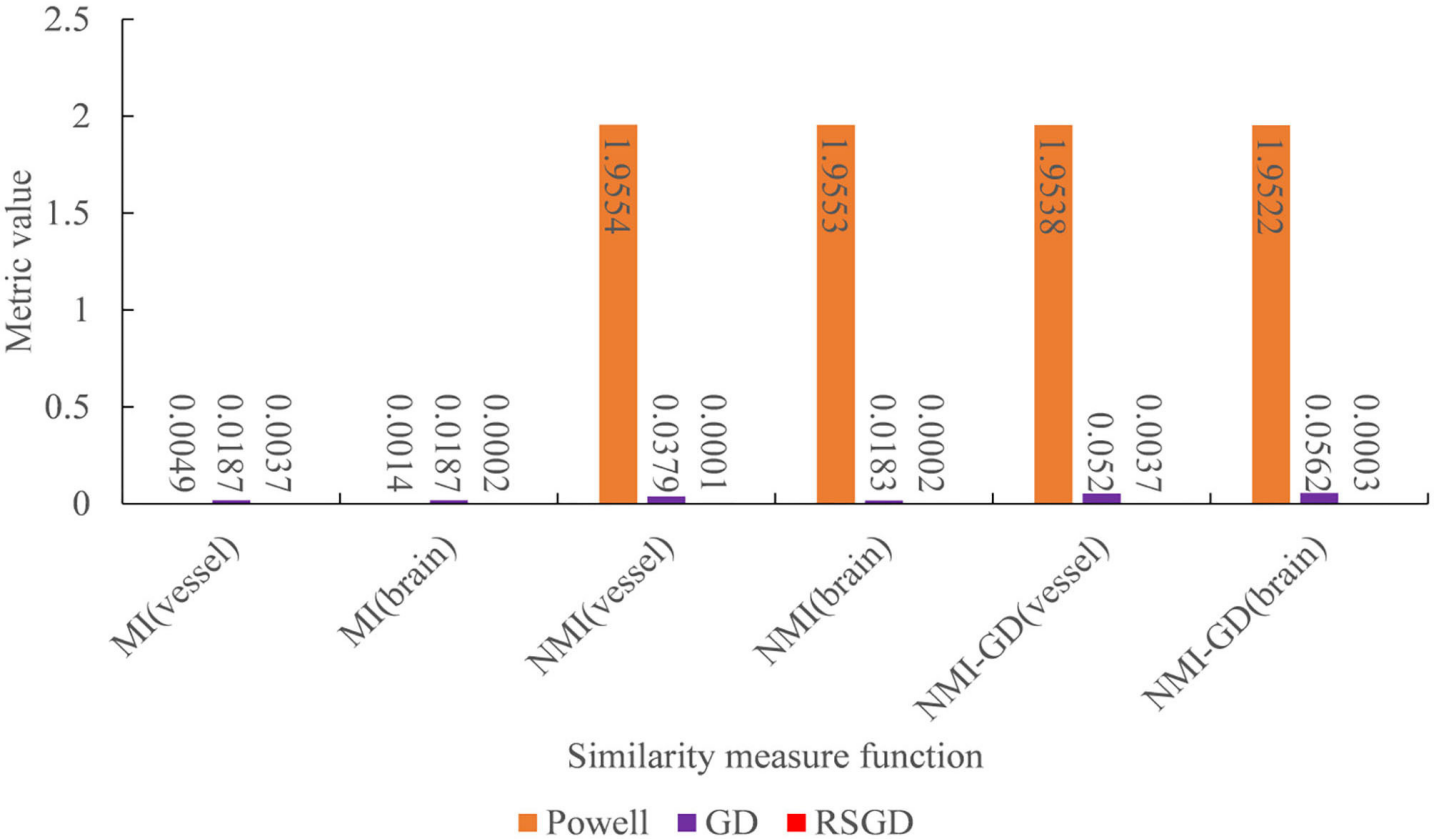
Under the condition of different similarity measure functions, the measure values of the three optimization algorithms.

**Figure 8:**
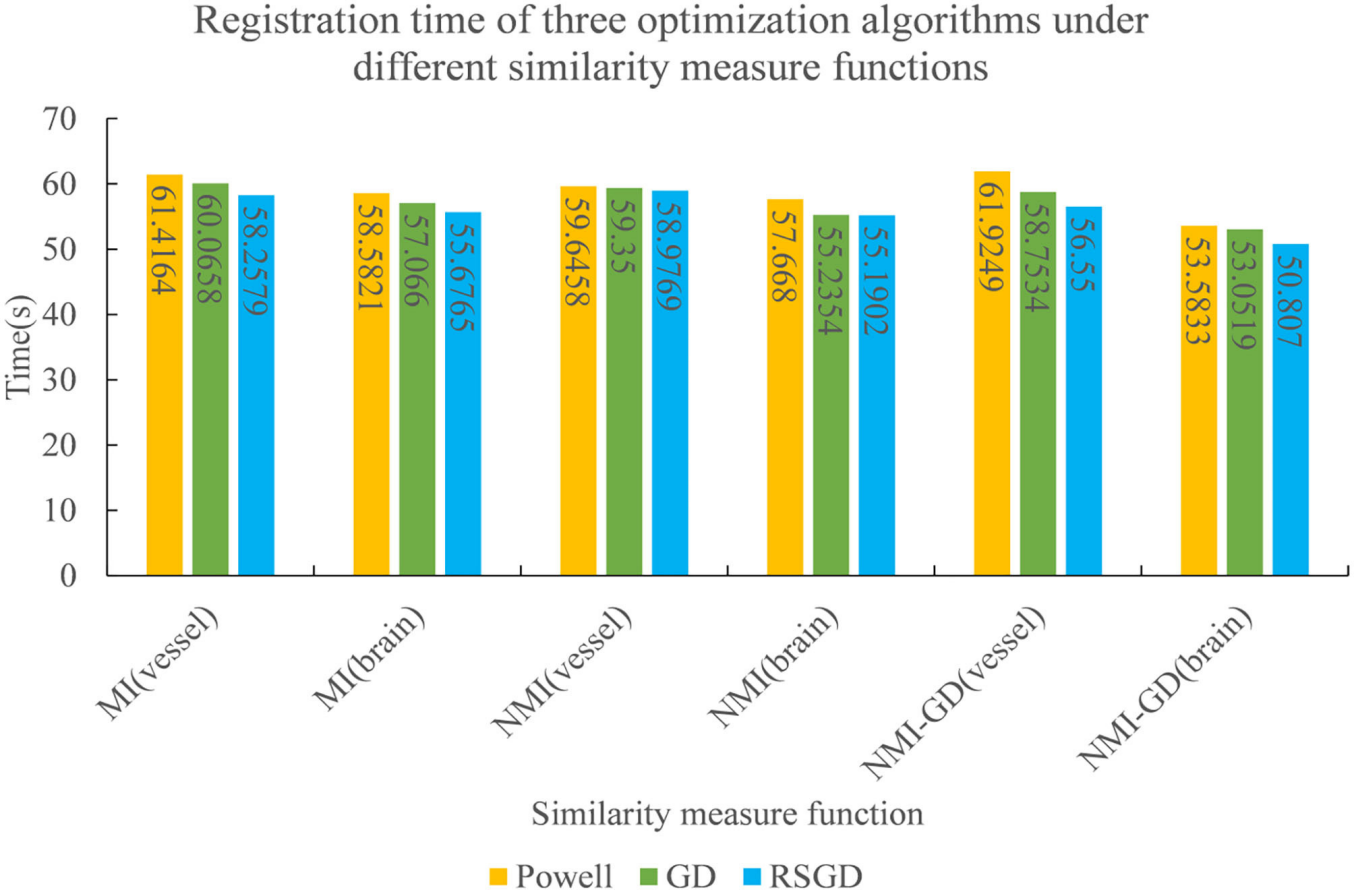
The registration time spent by the three optimization algorithms under the conditions of different similarity measure functions.

To verify the advantages of the weighting function and the optimization algorithm proposed in this study, two controlled experiments of three optimization algorithms (Powell, GD, and RSGD) under three similarity metric functions (MI, NMI, and NMG) were conducted on two sets of data, the blood vessels and the brain, and the comparative experimental results are shown in Figures 7, 8, analyzed by the qualitative and quantitative results obtained from the aforementioned sets of experiments, and the registration method proposed in this study shows certain advantages in registration efficiency and registration accuracy.

## 5. Discussion

During the intervention of cerebrovascular disease, accurately finding the vascular morphology of the target region will greatly improve the success rate of the operation. In the field of 2D–3D medical image registration, it is important to improve the registration accuracy and reduce the registration time to locate the lesion more accurately in surgical procedures. When multi-resolution completes sampling, the similarity is obtained by NMG and the optimal value is iteratively obtained by the RSGD algorithm, so that our method can achieve the effect of optimal registration, and the spatial position parameters of the CT image are continuously adjusted in this process, so that the projected image can be accurately aligned with the reference image.

According to the preliminary experiments and controlled experiments done on two groups of human data, the finally presented experimental data and registration results show that the registration method proposed in this experiment has a better performance for 2D–3D registration. The errors of spatial position parameters of the images in both sets of experiments are 0, and the spent registration time is also the smallest. The experimental results obtained from the control experiments after the initial spatial parameters are changed also meet the requirements. The traditional similarity metric functions, MI and NMI, contain only the grayscale information of the image, which leads to an increase in the error of the final registration results.

The fusion with the gradient difference function makes up for the lack of spatial feature information of the original algorithm in the image, and the NMG function proposed in this experiment can capture both grayscale and spatial information of the image. The multi-resolution strategy selects the pyramid convolution algorithm, which adds another convolution layer before each original pyramid layer. The Strategy can improve the sampling signal ability of the pyramid and play the role of suppressing image noise while improving the robustness of the algorithm. The optimization algorithm selects the RSGD, which avoids local extremes by changing the step length of gradient descent in each iteration process, and which can speed up the registration speed.

Based on the aforementioned analysis, the results and registration efficiency of the optimized NMG registration method using RSGD based on a multi-resolution sampling strategy for multimodal image registration are excellent compared with the other two optimization algorithms; however, this experiment can align only single images at present and cannot perform batch processing, which is the limitation of this experiment.

## 6. Conclusion

This study introduces the RSGD-optimized NMG registration method based on the multi-resolution strategy, while experiments show that the registration performance has improved greatly compared with the traditional registration method. NMG makes the aligned image contain grayscale information and spatial gradient information, while the multi-resolution strategy improves the image signal sampling ability and enhances the stability and robustness. RSGD can avoid local extremes to approach the optimal solution of the objective function faster. Future research prospects can look into non-rigid 2D–3D alignment algorithms and how to efficiently process large batches of aligned images.

## Data availability statement

The raw data supporting the conclusions of this article will be made available by the authors, without undue reservation.

## Ethics statement

The studies involving human participants were reviewed and approved by Shenzhen Second People's Hospital. The patients/participants provided their written informed consent to participate in this study.

## Author contributions

XZ: design and program implementation of experimental ideas and methods. YD: preparation of experimental materials. CT: visualization of experimental results. SC: arrangement of experimental data. YW: verification of experimental design. MZ: experimental data analysis. QW: review and revision of papers. XL: experimental supervision and leadership. WS: review and revision of the first draft of the papers. All authors contributed to the article and approved the submitted version.

## Appendix

(1)
$$\begin{array}{l} NMG\left(A,B\right)=\alpha_{1}\cdot I_{NMI}\left(A,B\right)+\alpha_{2}\cdot I_{GD}\left(A,B\right) \end{array}$$

(2)
$$\begin{array}{l} H\left(A\right)=-\overset{N}{\underset{i=1}{\sum }}p\left(A_{i}\right)\log_{p}\left(A_{i}\right) \end{array}$$

(3)
$$\begin{array}{l} H\left(A,B\right)=-\overset{N}{\underset{i=1}{\sum }}\overset{N}{\underset{j=1}{\sum }}p\left(A_{i},B_{j}\right)\log_{p}\left(A_{i},B_{j}\right) \end{array}$$

(4)
$$\begin{array}{l} MI\left(A,B\right)=H\left(A\right)+H\left(B\right)-H\left(A,B\right) \end{array}$$

(5)
$$\begin{array}{l} NMI\left(A,B\right)=\frac{H\left(A\right)+H\left(B\right)}{H\left(A,B\right)}=1+\frac{MI\left(A,B\right)}{H\left(A,B\right)} \end{array}$$

(6)
$$\begin{array}{l} GD\left(A,B\right)=\underset{m,n}{\sum }\frac{\sigma_{v}}{\sigma_{v}+{\left(I_{dV}\left(m,n\right)\right)}^{2}}+\underset{m,n}{\sum }\frac{\sigma_{h}}{\sigma_{h}+{\left(I_{dH}\left(m,n\right)\right)}^{2}} \end{array}$$

(7)
$$\begin{array}{l} I_{dV}\left(m,n\right)=\frac{dA}{dm}-s*\frac{dB}{dm} \end{array}$$

(8)
$$\begin{array}{l} I_{dH}\left(m,n\right)=\frac{dA}{dn}-s*\frac{dB}{dn} \end{array}$$

(9)
$$\begin{array}{l} g_{k}\left(i,j\right)=\underset{m}{\sum }\underset{n}{\sum }\left(W\left(m,n\right)\times g_{k-1}\left(2i+m,2j+n\right)\right) \end{array}$$

(10)
$$\begin{array}{l} \theta_{k+1}=\theta_{k}-\alpha_{k}d_{k} \end{array}$$
